# The New Texas Sanitary Law

**Published:** 1904-01

**Authors:** George R. Tabor

**Affiliations:** State Health Officer; Austin, Texas


### THE NEW TEXAS SANITARY LAW.

**Disinfection of Railway Coaches, Public Buildings and Sleeping Cars.**

The State Health Officer of Texas has just formulated the rules and regulations to govern the disinfection of sleeping cars and public buildings, as required by the law passed by the last Legislature, and the "Red Back" is the first to publish them. We give below the text of the law in order that our readers may understand and appreciate the vast importance of this sanitary reform, and the first draft of the rules to carry it into effect. The law became operative in July last, but because of his duties as Surgeon-General of Texas before, during and since the encampment of the Texas National Guard (the Texas Militia) and the responsible, onerous and long enduring work in the management of two yellow fever epidemics, and other duties of his office, Dr. Tabor really has not had time, nor has he the adequate assistance, to give this matter the attention its importance demands. These rules will be at once and rigidly enforced. They will be supplemented and amplified from time to time as the necessity for same becomes evident. Dr. Tabor will call a meeting in Austin of all county and city health officers some time in February, for a further conference as to details necessary to make this law effective. By "public buildings" is meant public schools, hospitals, jails, court houses, etc., but whether it means hotels or not, is yet to be decided by the Attorney General.

### DISINFECTION OF PUBLIC BUILDINGS, RAILWAY COACHES AND SLEEPING CARS.

S. B. No. 95.] CHAPTER CXIV.

An Act requiring the disinfection of public buildings, railway coaches and sleeping cars, and providing a penalty for the violation thereof, and declaring an emergency.

SECTION 1. *Be it enacted by the Legislature of the State of Texas:* That it shall be the duty of the State Health Officer of Texas, and he is hereby authorized and empowered to prepare rules and regulations governing the proper disinfection and sanitation of public buildings and all railway coaches and sleeping cars operated in the State of Texas.

SEC. 2. It shall be his duty and he is hereby authorized and empowered to prescribe a sanitary code, which shall contain and provide rules and regulations of a general nature for the improvement and amelioration of the hygienic and sanitary condition of said public buildings, railway coaches and sleeping cars.

SEC. 3. Every person having control of any public building, railway company, sleeping car company, or other corporation, company or individual, or the receiver thereof, engaged in the carrying of passengers in this State, shall, at their own expense, within a prescribed time after receiving notice from the State Health Officer of the promulgation of the rules and regulations in the above sections mentioned, carry the same into effect.

SEC. 4. If any person having control of any public building, or any agent, manager, operator, employe or receiver of any railway company, sleeping car company, or any individual shall fail to comply with the provisions of this act, and the rules and regulations promulgated by the State Health Officer under the provisions thereof, he shall be deemed guilty of a misdemeanor, and upon conviction shall be punished by a fine of not less than fifty nor more than two hundred dollars.

Approved April 6, 1903.

CIRCULAR NO. 1.

By virtue of the authority vested in me by the above act of the Twenty-eighth Legislature, the following rules are hereby prescribed, which shall govern the disinfection and sanitation of public buildings, railway coaches and sleeping cars in the State of Texas, and shall be effective on and after February 11, 1904:

1. Each passenger coach or sleeping car used for passengers must be provided with one cuspidor for each seat or every two chairs. Each cuspidor must contain not less than six ounces of a disinfectant solution approved by this department. The cuspidors to be emptied, washed in a similar solution, and replenished each trip or every twenty-four hours.

2. Public buildings must be provided with sufficient number of cuspidors, or not less than one in each room or hall, treated in a like manner and emptied, washed and replenished daily.

3. The floors of cars and public buildings must be sprinkled with a similar solution before each sweeping.

4. Sweeping and dusting of cars are prohibited in transit, except that floors of cars may be swept at division terminals or meal stations where passengers will be given an opportunity to leave the cars during that time. Seats, windows and walls of cars must be wiped off with a cloth or sponge and not dusted in transit.

5. All sleeping cars must be disinfected by fumigation in a manner approved by this department at the end of each round trip in the State of Texas where sleeping cars do not leave the State.

6. All sleeping cars passing through or coming into the State of Texas must be disinfected in the same manner each trip at some point in the State approved by this department. All carpets, curtains, blankets and bedding, except linen, to be disinfected with cars.

7. Day coaches used for passengers must be fumigated, whenever the necessity exists, at some point in this State acceptable to this department. If a car becomes infected by being occupied by a person having a contagious disease it must be disinfected immediately at end of run.

8. All public buildings must be disinfected by fumigation whenever the necessity exists for it.

9. Containers of water for drinking, in cars and public buildings, must be emptied and thoroughly cleansed at least once every forty-eight hours. (Public schools should be provided with a separate cup at each desk for each pupil to drink from, or the pupils should be required to provide same.)

10. Ice, which is used in water coolers in cars, must not be dumped on the floors, sidewalks and car platforms where people have trod and expectorated, and then picked up by unclean hands and put into the drinking water. It should be washed and handled with ice tongs.

11. Passengers, patrons, employes or others must be prohibited from washing their teeth over or expectorating in basins in sleeping cars, passenger coaches or public buildings which are used for bathing the face and hands. Large cuspidors must be provided for such purposes.

All local health officers and citizens are requested to assist in the enforcement of the above rules.

(Signed) GEORGE R. TABOR,
State Health Officer.

Austin, Texas, January 11, 1904.

